# Is Recurrent Endometriosis a Reprogrammed Disease? Molecular Persistence Beyond Surgical Clearance

**DOI:** 10.3390/cells15100951

**Published:** 2026-05-21

**Authors:** Mario Palumbo, Luigi Della Corte, Maria Rotonda Conte, Giuseppe D’Angelo, Mario Ascione, Antonisia Pollio, Pierluigi Giampaolino, Giuseppe Bifulco

**Affiliations:** 1 Department of Public Health, School of Medicine, University of Naples “Federico II”, 80131 Naples, Italy; conte.maria128@gmail.com (M.R.C.); giuseppe.dangelo@unina.it (G.D.); mario.ascione@unina.it (M.A.); antonisiapollio@gmail.com (A.P.); pierluigi.giampaolino@unina.it (P.G.); giuseppe.bifulco@unina.it (G.B.); 2 Department of Neuroscience, Reproductive Sciences and Dentistry, School of Medicine, University of Naples “Federico II”, 80131 Naples, Italy; luigi.dellacorte@unina.it

**Keywords:** endometriosis, molecular memory, epigenetics, cellular reprogramming, inflammatory microenvironment, immune escape

## Abstract

**Background:** Endometriosis is traditionally conceptualized as a localized gynecological disorder characterized by the presence of ectopic endometrial tissue. However, high recurrence rates following apparently complete surgical excision challenge this lesion-based paradigm and suggest the existence of underlying biological mechanisms that extend beyond residual disease. Increasing evidence indicates that endometriotic cells exhibit persistent molecular alterations, including dysregulated gene expression, epigenetic modifications, and immune dysfunction, which may contribute to disease maintenance and recurrence. **Objective:** This study aims to critically examine whether endometriosis can be considered a molecularly reprogrammed disease, characterized by persistent cellular and microenvironmental alterations that are not reversed by surgical removal of visible lesions. **Methods:** A narrative review of the literature was conducted using PubMed, Scopus, and Web of Science databases including studies published from January 2016 to March 2026. Studies investigating molecular, genetic, epigenetic, and immunological mechanisms of endometriosis persistence and recurrence were included. Particular attention was given to pathways involved in cellular survival, inflammation, hormone resistance, and epigenetic regulation. **Results:** Endometriotic cells demonstrate stable alterations in gene expression profiles, including pathways related to estrogen signaling, progesterone resistance, inflammation, and cellular proliferation. Epigenetic mechanisms, such as aberrant DNA methylation and histone modifications, appear to sustain these changes over time, contributing to a form of “molecular memory.” In parallel, the peritoneal microenvironment is characterized by chronic inflammation, immune tolerance, and impaired clearance of ectopic cells. These factors collectively support lesion persistence and may explain recurrence even after complete surgical excision. Emerging evidence also highlights the role of systemic factors, including endocrine–immune interactions and microbiome-related pathways, reinforcing the concept of endometriosis as a systemic rather than purely localized condition. **Conclusions:** Endometriosis may be more accurately defined as a persistent, molecularly reprogrammed disease driven by stable alterations in cellular behavior and the surrounding microenvironment. This paradigm shift has important clinical implications, suggesting that surgical treatment alone may be insufficient and that future therapeutic strategies should target the underlying molecular and immunological mechanisms responsible for disease persistence.

## 1. Introduction

Endometriosis is a chronic, estrogen-dependent inflammatory disease characterized by the presence of endometrial-like tissue outside the uterine cavity, affecting approximately 10% of women of reproductive age and representing a major cause of chronic pelvic pain, infertility, and reduced quality of life [1,2,3,4,5]. Traditionally, the disease has been conceptualized as a localized gynecological condition, in which ectopic lesions are identified and surgically removed with the aim of achieving symptom resolution and disease control [6,7,8].

Despite significant advances in surgical techniques and imaging modalities, recurrence remains a major clinical challenge. A substantial proportion of patients experience symptom relapse and/or lesion reappearance even after apparently complete surgical excision performed by experienced surgeons. While recurrence has historically been attributed to incomplete removal of microscopic lesions or de novo lesion formation, these explanations do not fully account for the high rates of relapse observed in clinical practice, particularly following optimal surgical clearance [9,10,11,12,13].

In recent years, increasing attention has shifted toward the intrinsic biological properties of endometriotic cells and their interaction with the surrounding microenvironment. Accumulating molecular evidence indicates that endometriotic tissue represents a distinct cellular entity rather than a simple ectopic counterpart of eutopic endometrium. These cells exhibit stable alterations in gene expression, progesterone resistance, enhanced proliferative and invasive capacity, and resistance to apoptosis, supported by dysregulation of key signaling pathways, including estrogen receptor signaling, inflammatory cascades, and intracellular networks such as Wnt/β-catenin and PI3K/AKT/mTOR [14,15,16,17,18].

Epigenetic regulation has emerged as a central mechanism underlying these persistent alterations. Aberrant DNA methylation, histone modifications, and non-coding RNA expression contribute to long-lasting transcriptional reprogramming without changes in DNA sequence. These mechanisms may establish a form of “molecular memory,” enabling endometriotic cells to maintain a pathological phenotype over time, even in the absence of the original initiating stimuli [19,20,21,22].

Beyond intrinsic cellular changes, the peritoneal and systemic microenvironment plays an active and dynamic role in disease persistence. Endometriosis is characterized by a chronic inflammatory milieu, with increased levels of pro-inflammatory cytokines, altered immune surveillance, and enhanced angiogenesis. Impaired immune-mediated clearance of ectopic cells, together with increased immune tolerance, contributes to the survival and re-establishment of lesions after surgical removal [23,24,25,26]. These processes occur within a complex network of interactions between reprogrammed cells and a permissive biological environment, further reinforced by systemic factors [27,28,29,30].

Collectively, these observations challenge the traditional lesion-based paradigm and support the concept that endometriosis extends beyond a purely localized disorder, involving persistent molecular and microenvironmental alterations that are not eliminated by surgery alone [31,32]. This perspective provides a mechanistic explanation for recurrence and highlights the limitations of approaches focused exclusively on anatomical disease [33,34].

While recent comprehensive reviews have emphasized the systemic nature of endometriosis, the present work introduces the concept of stable cellular reprogramming as a unifying framework linking epigenetic molecular memory, microenvironmental persistence, and clinical recurrence [35,36,37,38]. Importantly, this review further integrates these mechanisms with reproductive biology, highlighting how molecular reprogramming directly impacts endometrial receptivity, gamete quality, and reproductive outcomes, an aspect that remains insufficiently addressed in previous systemic models.

This narrative review was conducted through a literature search performed using PubMed, Scopus, and Web of Science databases. Studies published between January 2016 and March 2026 were considered. Priority was given to translational, molecular, immunological, epigenetic, and clinically relevant studies addressing endometriosis persistence, recurrence, hormonal resistance, inflammatory pathways, immune dysfunction, and fertility implications.

Based on this framework, the present review aims to examine endometriosis as a molecularly reprogrammed disease characterized by persistent alterations in cellular behavior and tissue environment. We specifically analyze the roles of genetic susceptibility, epigenetic regulation, inflammatory signaling, and immune dysfunction in sustaining disease activity beyond surgical clearance, with particular emphasis on their implications for fertility and reproductive outcomes. By reframing endometriosis within this integrated biological paradigm, this work seeks to provide a foundation for the development of more effective, mechanism-based and personalized therapeutic strategies.

In addition, it is important to recognize that disease persistence in endometriosis does not exclusively manifest as macroscopic recurrence. A substantial proportion of patients continue to experience pain and functional impairment even in the absence of detectable lesions at imaging or surgery. This suggests that persistent biological activity, including microscopic foci, altered cellular behavior, and ongoing inflammatory signaling, may contribute to symptom generation independently of overt anatomical disease.

Given the narrative nature of the review, studies were selected based on relevance, translational value, methodological quality, and conceptual contribution to the emerging model of endometriosis as a biologically sustained disease.

## 2. Why Surgery Fails: Beyond Incomplete Lesion Removal

### 2.1. The Limits of a Lesion-Based Surgical Paradigm

In recent years, the management of endometriosis has progressively evolved from a predominantly surgery-centered approach toward a more individualized and phenotype-adapted strategy. While surgical excision remains a fundamental therapeutic option in selected patients, particularly in cases of moderate-to-severe disease, organ involvement, infertility, or failure of medical therapy, increasing awareness of recurrence risk, potential surgical morbidity, and the chronic nature of the disease has reinforced the role of long-term medical management in many clinical scenarios. In many patients, especially those with pain-predominant disease and without immediate surgical indications, medical therapy is currently recommended as first-line management. Within this context, the primary objective of surgery remains the removal of visible endometriotic lesions, restoration of pelvic anatomy, and improvement of symptoms and reproductive outcomes [39,40,41,42]. Advances in surgical techniques and high-resolution imaging have significantly improved the ability to identify and treat complex disease presentations.

Despite these improvements, recurrence rates remain clinically relevant, ranging from approximately 20% to 50% within five years depending on disease phenotype, surgical approach, postoperative management, and duration of follow-up [43,44,45,46]. Although incomplete excision, particularly in the presence of microscopic or deeply infiltrating lesions, undoubtedly contributes to recurrence in a proportion of cases, it may not entirely account for the heterogeneity of long-term clinical outcomes observed after apparently adequate surgical treatment [46,47,48,49]. 

Importantly, high-quality surgical excision performed by experienced surgeons has been consistently associated with improved symptom control and reduced recurrence risk, as emphasized in current international guidelines [44,50,51,52,53]. Nevertheless, the persistence or recurrence of symptoms and/or disease in a subset of patients suggests that anatomical removal alone may not always be sufficient to ensure durable long-term control [46,47,48,49].

Taken together, these observations highlight some limitations of a purely lesion-based paradigm [50,51,52,53,54]. While surgery effectively addresses the macroscopic manifestations of the disease, it may not fully modify the underlying biological mechanisms potentially contributing to disease persistence in certain patients. Within this perspective, endometriosis may extend beyond a purely structural disorder and, at least in a subset of individuals, may involve persistent molecular, inflammatory, immune, and microenvironmental alterations that are not completely reversed by surgical excision alone [55,56].

### 2.2. Microscopic Disease Versus Biological Persistence

The presence of microscopic or “occult” endometriosis has long been proposed as a key explanation for recurrence. Histological studies have demonstrated endometriotic implants in macroscopically normal peritoneum, suggesting that undetected lesions may contribute to disease re-establishment [51,52,53]. However, the clinical relevance of these findings remains inconsistent, as the presence of microscopic foci does not reliably correlate with symptom recurrence or disease progression [33,54].

An alternative and increasingly supported interpretation is that recurrence reflects biological persistence rather than purely anatomical residual disease. Endometriotic cells exhibit intrinsic properties distinct from eutopic endometrium, including enhanced survival, resistance to apoptosis, and the ability to proliferate, adhere, and invade ectopic sites [14,15,16,17,18]. These features are maintained by stable molecular and epigenetic alterations, allowing cells to retain a pathological phenotype over time [19,20,21,22].

Within this framework, even a small number of residual or newly implanted cells may be sufficient to reinitiate disease in a permissive microenvironment [10,11,12,13]. Thus, recurrence is more accurately understood as the result of persistent cellular programming interacting with a supportive biological context, rather than the mere presence of undetected lesions.

Importantly, biological persistence may not always translate into detectable lesion recurrence. In some patients, residual or newly established microscopic foci, together with sustained inflammatory and neurogenic signaling, may be sufficient to maintain symptoms even in the absence of macroscopic disease. This may explain the well-recognized clinical scenario of persistent pain despite apparently complete surgical excision.

### 2.3. Molecular Reprogramming and Resistance to Clearance

A potentially important mechanism contributing to disease persistence in endometriosis is the concept of molecular reprogramming. Increasing evidence suggests that, in at least a subset of patients, endometriotic cells may exhibit relatively stable alterations in gene expression involving pathways related to estrogen signaling, inflammatory responses, cellular survival, and tissue remodeling. In particular, progesterone resistance combined with enhanced estrogen responsiveness may contribute to the establishment of a hormonal environment favoring sustained proliferation, impaired differentiation, and reduced responsiveness to physiological regulatory mechanisms [57].

These alterations appear to be influenced, at least in part, by epigenetic regulatory mechanisms, including DNA methylation, histone modifications, and dysregulated non-coding RNA expression. Through these processes, endometriotic cells may acquire relatively persistent transcriptional profiles that could contribute to the maintenance of pathological cellular behavior over time, a phenomenon that has been described as a form of “molecular memory” [19,20,21,22,58].

Functionally, this altered cellular state has been associated with reduced susceptibility to apoptosis, increased adaptability to inflammatory and hypoxic microenvironments, and enhanced proliferative and invasive potential [14,15,16,17,18]. In parallel, dysregulation of signaling pathways such as PI3K/AKT/mTOR and Wnt/β-catenin may further support cellular survival, inflammatory activation, and resistance to therapeutic control [26,27,28].

Within this framework, surgical excision effectively removes the macroscopic anatomical manifestations of disease but may not fully reverse the biological alterations potentially present in residual ectopic cells, microscopic foci, or even the eutopic endometrium in certain patients. Consequently, persistent or newly implanted cells carrying these altered molecular characteristics may contribute to disease persistence or recurrence over time, providing a possible mechanistic link between molecular dysregulation and heterogeneous long-term clinical outcomes [56,57,58,59,60,61].

### 2.4. The Role of the Inflammatory and Immune Microenvironment

In addition to intrinsic cellular alterations, the peritoneal microenvironment plays a central role in disease persistence. Endometriosis is characterized by a chronic inflammatory state, with elevated levels of cytokines such as interleukin-6, tumor necrosis factor-α, and interleukin-1β, which promote angiogenesis, cellular proliferation, and nociceptive signaling [23,24,25,26,27]. This inflammatory milieu not only sustains lesion survival but also contributes to symptom progression, particularly pain sensitization [29,30].

Beyond cytokine overproduction, persistent activation of inflammatory signaling pathways, most notably NF-κB-further amplifies this process, promoting the transcription of genes involved in cell survival, adhesion, and immune modulation. In parallel, increased oxidative stress and the accumulation of reactive oxygen species (ROS) contribute to DNA damage, mitochondrial dysfunction, and the maintenance of a pro-inflammatory environment, reinforcing lesion persistence.

Immune dysfunction further consolidates this permissive niche. Under physiological conditions, ectopic endometrial cells should be effectively cleared by immune surveillance; however, in endometriosis, this process is impaired [62,63,64,65]. Reduced natural killer (NK) cell cytotoxicity, altered T-cell responses with expansion of regulatory T cells, and macrophage polarization toward pro-repair and pro-angiogenic phenotypes collectively support cellular survival and implantation [66,67,68,69]. Notably, these immune alterations coexist with chronic inflammation, reflecting a paradoxical state in which inflammatory activation does not translate into effective clearance of ectopic tissue.

Importantly, these changes are not transient and may persist even after surgical removal of visible lesions. The maintenance of a dysregulated inflammatory and immune environment creates a stable biological niche that facilitates the survival, implantation, and reactivation of endometriotic cells, thereby contributing to disease recurrence [37,38]. Within this framework, the microenvironment should be regarded not as a passive background, but as an active driver of disease persistence, tightly interacting with the intrinsic reprogramming of endometriotic cells.

Taken together, these findings support the concept that endometriosis may be more accurately defined as a systemic inflammatory disease.

The persistence of a dysregulated inflammatory and immune microenvironment may also support the activity of minimal or microscopic cellular clusters that are not detectable by conventional imaging or surgical inspection, yet remain biologically active. These microfoci may contribute disproportionately to symptom generation, particularly pain, through neuroinflammatory mechanisms.

### 2.5. Systemic and Non-Local Drivers of Recurrence

Increasing evidence suggests that, at least in a subset of patients, endometriosis may involve biological regulatory mechanisms extending beyond the local peritoneal environment [31,32,35]. Alterations in endocrine, immune, inflammatory, and metabolic pathways may contribute to a broader systemic context capable of sustaining disease activity and potentially influencing the marked heterogeneity observed in clinical presentation, symptom severity, recurrence risk, and treatment response [70,71,72].

In particular, dysregulation of the hypothalamic–pituitary–ovarian (HPO) axis may contribute to the persistence of an estrogen-dominant milieu characterized by enhanced estrogen signaling and impaired progesterone responsiveness. Importantly, this hormonal imbalance does not act in isolation but appears to interact closely with inflammatory and immune pathways. Chronic low-grade systemic inflammation may amplify local inflammatory processes through persistent cytokine production, immune cell recruitment, oxidative stress, and tissue remodeling within the peritoneal environment [22,23,24,25,73].

At the same time, metabolic and immunological alterations may contribute to a complex and dynamic network of systemic regulation. Emerging evidence suggests that endometriosis may be associated with subtle but persistent changes in immune function, including altered cytokine profiles, macrophage activity, and immune cell signaling at both local and systemic levels. Although the directionality of these alterations remains incompletely understood, such changes may facilitate the survival of ectopic cells and support their ability to adapt to different biological environments [37,38].

Within this framework, the gut microbiome has also gained increasing attention as a potential modulator of disease activity. Dysbiosis may influence immune responses, inflammatory signaling, and estrogen metabolism through modulation of the estrobolome, thereby indirectly sustaining estrogen-dependent proliferation and chronic inflammation [40,41,42]. Although causal relationships remain to be fully established, these observations suggest that host–microbiome interactions may represent an additional layer of biological regulation contributing to disease persistence in some patients [42,43,44].

Interestingly, indirect support for the existence of persistent non-local biological drivers also emerges from current postoperative management strategies. In routine clinical practice, long-term hormonal suppression is frequently recommended after surgical excision in order to reduce recurrence risk and maintain symptom control, particularly in patients not immediately seeking pregnancy. While this approach does not demonstrate the existence of a universally systemic disease process, it nevertheless suggests that surgery alone may not always be sufficient to fully suppress the biological mechanisms contributing to ongoing disease activity in certain individuals. In this context, postoperative medical therapy may be interpreted not only as prevention of residual lesion growth, but also as an attempt to modulate the persistent hormonal, inflammatory, and microenvironmental conditions that may continue to support disease reactivation over time [45].

Taken together, these observations suggest that endometriosis may not be fully explained by local anatomical mechanisms alone. Rather, at least in a subset of patients, the disease may involve interconnected biological networks integrating endocrine, immune, inflammatory, metabolic, and microbial factors. This perspective may help explain why surgical excision, although highly effective for removing visible lesions and restoring pelvic anatomy, does not always guarantee durable long-term disease control. Indeed, surgery primarily targets the anatomical manifestations of the disease, whereas some of the underlying biological processes potentially contributing to persistence and recurrence may remain partially active even after apparently complete macroscopic excision.

### 2.6. Toward a New Conceptual Framework

Taken together, the evidence discussed in the previous sections indicates that the failure of surgery in endometriosis cannot be explained solely by technical limitations or incomplete lesion removal. While residual microscopic disease undoubtedly contributes to recurrence in a subset of patients, it does not fully account for the persistence and re-establishment of lesions observed even after apparently optimal surgical excision [14,15,16,17,18].

A more comprehensive interpretation emerges when considering the interplay between intrinsic cellular alterations and the surrounding biological environment. Endometriotic cells exhibit stable molecular and epigenetic reprogramming, enabling them to maintain a pathological phenotype characterized by enhanced survival, proliferation, and resistance to apoptosis. At the same time, the peritoneal microenvironment, marked by chronic inflammation, immune tolerance, and angiogenic activity, provides a permissive niche that supports lesion persistence and re-initiation [29,30,31,32].

Importantly, these local processes are further reinforced by systemic regulatory factors, including endocrine imbalance, immune dysregulation, and metabolic influences. The integration of these components results in a multi-level network in which cellular, microenvironmental, and systemic drivers interact dynamically to sustain disease activity over time. Within this framework, recurrence is not simply the consequence of residual lesions, but rather the expression of an underlying and persistent biological state.

This perspective allows surgical intervention to be more accurately contextualized. Surgery effectively addresses the anatomical manifestations of endometriosis, removing visible lesions and temporarily restoring pelvic anatomy. However, it does not directly modify the molecular and systemic mechanisms that drive disease persistence. As a result, the biological conditions that favor lesion development may remain active even after complete macroscopic clearance, enabling residual or newly implanted cells to re-establish disease.

Recognizing endometriosis as a biologically sustained condition has important implications for both research and clinical practice. It highlights the need to move beyond a purely lesion-centered paradigm and to adopt integrated therapeutic strategies targeting molecular reprogramming, immune dysfunction, and inflammatory signaling. Such an approach may be essential to achieve durable disease control and to reduce recurrence, particularly in patients with aggressive phenotypes or early-onset disease [34,45,46,47].

## 3. Molecular Reprogramming of Endometriotic Cells

### 3.1. Endometriotic Cells as a Distinct Biological Entity

Endometriotic lesions have traditionally been interpreted as ectopic counterparts of the eutopic endometrium, most commonly explained through retrograde menstruation and subsequent implantation of endometrial cells outside the uterine cavity. However, increasing molecular and functional evidence suggests that, at least in a subset of patients, endometriotic cells may exhibit biological characteristics that extend beyond simple ectopic displacement [47,48].

Comparative transcriptomic, proteomic, and functional studies have identified alterations involving pathways related to estrogen signaling, inflammatory responses, extracellular matrix remodeling, cellular adhesion, angiogenesis, and tissue invasion [14,15,16,17,18,74]. These molecular changes have been associated with enhanced cellular survival, reduced susceptibility to apoptosis, increased invasive potential, and greater adaptability to ectopic environments. Functional studies have also demonstrated altered adhesion molecule expression, increased matrix metalloproteinase activity, and enhanced migratory behavior, potentially facilitating implantation and tissue infiltration.

In parallel, evidence of metabolic reprogramming, including a shift toward glycolytic metabolism, altered mitochondrial function, and adaptation to oxidative stress, further supports the ability of endometriotic cells to survive under inflammatory and hypoxic conditions [75]. Collectively, these features suggest the acquisition of a relatively persistent pathological phenotype that may favor lesion establishment and long-term maintenance in ectopic sites.

Importantly, some of these molecular and epigenetic alterations have also been described, although generally to a lesser extent, in the eutopic endometrium of women with endometriosis. This observation raises the possibility that, in certain patients, at least part of the altered cellular behavior may precede ectopic implantation and reflect an intrinsic predisposition rather than being solely a consequence of the ectopic microenvironment [15,16,35,36]. At the same time, the more pronounced molecular alterations typically observed in ectopic lesions suggest that interactions with inflammatory, hormonal, immune, and microenvironmental factors likely contribute to amplification and stabilization of the pathological phenotype after implantation.

Within this framework, endometriosis may be interpreted not simply as passive ectopic implantation of otherwise normal endometrial tissue, but rather as a heterogeneous condition in which intrinsically altered cellular characteristics and permissive environmental factors interact dynamically to sustain disease development and persistence.

### 3.2. Hormonal Dysregulation and Progesterone Resistance

A defining feature of endometriotic cells is their altered responsiveness to steroid hormones, characterized by enhanced estrogen signaling and progesterone resistance [76].

At the molecular level, overexpression of estrogen receptor β (ERβ), often accompanied by a relative reduction in ERα expression, shifts estrogen signaling toward a pro-inflammatory and pro-survival profile. Increased local estrogen production, driven by aberrant aromatase activity and impaired estradiol metabolism, further amplifies this effect [76,77,78].

Conversely, progesterone receptor (PGR) expression, particularly the PR-B isoform, is significantly reduced in endometriotic tissue, leading to impaired downstream signaling [79]. This progesterone resistance disrupts normal anti-proliferative and anti-inflammatory responses, resulting in defective decidualization and sustained cellular proliferation.

These alterations are not merely functional but are underpinned by stable regulatory mechanisms. Epigenetic silencing of progesterone-related genes and persistent activation of estrogen-responsive pathways contribute to a long-lasting hormonal imbalance. This sustained dysregulation reinforces the reprogrammed phenotype of endometriotic cells and contributes to both lesion persistence and impaired endometrial receptivity.

### 3.3. Epigenetic Mechanisms and Molecular Memory

Epigenetic regulation is increasingly recognized as an important mechanism potentially contributing to the persistence of the endometriotic phenotype. DNA methylation, histone modifications, and non-coding RNAs collectively influence gene expression without altering the underlying DNA sequence and may contribute to relatively stable and heritable transcriptional changes over time [19,20,21,22]. Through these mechanisms, endometriotic cells may acquire altered regulatory programs associated with cellular survival, proliferation, inflammatory activation, and reduced responsiveness to physiological control signals.

Genome-wide methylation and transcriptomic studies have identified aberrant epigenetic patterns affecting genes involved in hormone signaling, inflammation, decidualization, and endometrial receptivity. Among the most well-characterized alterations, hypermethylation of the HOXA10 promoter has been associated with reduced gene expression in endometriotic endometrium.

This alteration results in impaired decidualization and reduced implantation capacity [80]. Similar epigenetic changes have been described for other progesterone-responsive genes, supporting the idea that epigenetic silencing contributes directly to progesterone resistance.

In parallel, epigenetic modulation of estrogen receptor-related genes, including alterations involving ESR2 regulation, may contribute to the altered ERβ/ERα balance observed in endometriotic tissue [21]. This imbalance has been associated with enhanced inflammatory signaling, increased cellular survival, and amplification of estrogen-dependent pathways. Histone modifications, through their effects on chromatin accessibility, together with dysregulated microRNAs and long non-coding RNAs, provide an additional layer of regulatory complexity capable of influencing multiple interconnected biological pathways.

Importantly, these epigenetic alterations likely do not occur in isolation but rather emerge through continuous interaction with the surrounding microenvironment. Inflammatory cytokines, oxidative stress, hypoxia, and hormonal exposure may influence epigenetic regulatory enzymes and progressively reinforce altered transcriptional states over time. This bidirectional interaction between environmental stimuli and epigenetic regulation may contribute to stabilization of disease-related biological pathways [21,80,81,82,83].

Within this context, the concept of “molecular memory” has been proposed to describe the possibility that endometriotic cells may retain persistent transcriptional and functional alterations following prior inflammatory, hormonal, or environmental exposures. Although the stability and reversibility of these mechanisms remain incompletely understood, accumulating evidence suggests that at least some epigenetic changes may persist even after apparent removal of the initiating stimuli or surgical excision of visible lesions. Taken together, these observations support the hypothesis that epigenetic dysregulation may contribute to disease persistence and heterogeneous clinical behavior in endometriosis. At the same time, they highlight the limitations of therapeutic approaches directed exclusively toward anatomical disease and suggest that long-term disease control may eventually require strategies capable of modulating the underlying regulatory networks sustaining pathological cellular behavior.

### 3.4. Activation of Pro-Survival and Proliferative Signaling Pathways

Endometriotic cells exhibit sustained activation of intracellular signaling pathways that promote survival, proliferation, and resistance to apoptosis. Among the most consistently implicated are the Wnt/β-catenin, PI3K/AKT/mTOR, and TGF-β signaling pathways [26,27,28]. These pathways play a central role in shaping the biological behavior of endometriotic cells, supporting their ability to persist and expand in ectopic environments.

Activation of the PI3K/AKT/mTOR pathway has been demonstrated in both human tissue samples and experimental models, with increased levels of AKT phosphorylation observed in endometriotic lesions compared to eutopic endometrium [82]. This signaling axis is closely linked to enhanced cell survival, angiogenesis, and metabolic adaptation, particularly under conditions of cellular stress such as hypoxia and inflammation. Its activation also contributes to resistance to apoptotic stimuli, allowing endometriotic cells to evade physiological mechanisms of clearance.

Similarly, aberrant activation of Wnt/β-catenin signaling has been associated with increased cellular invasiveness and impaired differentiation. This pathway influences the expression of genes involved in adhesion, migration, and extracellular matrix interaction, thereby facilitating implantation and tissue infiltration. In parallel, TGF-β signaling plays a key role in fibrosis, extracellular matrix remodeling, and immune modulation, processes that are particularly relevant in more advanced forms of the disease, including deep infiltrating endometriosis [75,81,82,83].

Importantly, these pathways do not function in isolation but are part of a highly interconnected signaling network. Crosstalk between these systems amplifies downstream effects and contributes to the stabilization of a pro-survival cellular phenotype. Inflammatory mediators and hormonal signals further modulate these pathways, creating a dynamic environment in which multiple regulatory inputs converge to sustain disease activity.

The sustained activation of these signaling networks enables endometriotic cells to adapt to adverse conditions, including hypoxia, oxidative stress, and chronic inflammation. As a result, these cells maintain their proliferative and invasive capacity over time and show a reduced response to therapeutic interventions. This persistent signaling activity provides a mechanistic link between molecular alterations and clinical disease behavior, reinforcing the concept of endometriosis as a condition driven by stable and self-maintaining biological processes.

### 3.5. Inflammatory Reprogramming and Immune Escape

Endometriotic cells exhibit a persistent pro-inflammatory phenotype that contributes to the establishment of a self-sustaining inflammatory network. Increased production of cytokines such as interleukin-6 and tumor necrosis factor-α activates key transcriptional pathways, including NF-κB, which in turn amplifies inflammatory signaling and promotes lesion survival [23,24,25,26,27]. This persistent activation is not merely a downstream consequence of inflammation but actively shapes cellular behavior, reinforcing proliferation, resistance to apoptosis, and local tissue invasion.

At the same time, endometriotic cells interact dynamically with the immune microenvironment, promoting conditions that favor immune evasion. Under physiological circumstances, ectopic endometrial cells should be efficiently cleared through immune surveillance. However, in endometriosis, this process is compromised. Reduced susceptibility to natural killer (NK) cell-mediated cytotoxicity, together with increased regulatory T-cell activity and altered macrophage polarization, contributes to the establishment of a state of immune tolerance [65,66,67,68]. These changes limit effective immune clearance while allowing inflammatory signaling to persist.

Macrophages within endometriotic lesions frequently display a phenotype that supports angiogenesis, tissue remodeling, and cytokine production rather than efficient phagocytic activity. This functional shift not only sustains inflammation but also promotes vascularization and structural stabilization of lesions. In parallel, altered communication between immune cells and endometriotic tissue further reinforces a permissive microenvironment in which pathological processes are maintained rather than resolved.

A notable feature of this context is the coexistence of chronic inflammation and ineffective immune response. Rather than leading to resolution, inflammation becomes part of a dysregulated feedback system in which immune activation and immune escape occur simultaneously. This imbalance contributes to the persistence of ectopic cells and facilitates their ability to adapt and survive over time.

Within this framework, inflammatory reprogramming and immune escape emerge as tightly interconnected processes. Together, they enable endometriotic cells to survive in ectopic environments, resist host defenses, and re-establish disease even after surgical removal of lesions. These mechanisms provide a direct link between molecular alterations and clinical recurrence, further supporting the concept of endometriosis as a condition driven by stable and self-perpetuating biological processes [33,34,35,36].

### 3.6. Integration of Molecular Pathways: Toward a Reprogrammed Disease Model

The molecular alterations described above, including hormonal dysregulation, epigenetic reprogramming, activation of pro-survival signaling pathways, and immune modulation, should be interpreted as interconnected components of a unified biological system rather than isolated mechanisms [14,15,16,17,18,31,32]. These processes interact continuously, with each layer influencing and reinforcing the others, ultimately shaping the overall behavior of endometriotic cells.

Within this context, endometriotic cells exist in a relatively stable reprogrammed state in which multiple regulatory networks converge to maintain a pathological phenotype. Hormonal imbalance, epigenetic changes, and intracellular signaling pathways are closely intertwined with inflammatory and immune processes, creating a dynamic yet self-sustaining system. This integration allows cells not only to survive but also to adapt to different environmental conditions, including hypoxia, oxidative stress, and fluctuating hormonal signals. (Table 1).

Importantly, these interactions give rise to self-reinforcing feedback loops that stabilize disease-related pathways over time. For example, inflammatory mediators can further modulate epigenetic regulators and signaling cascades, while altered hormonal signaling may amplify both inflammatory and proliferative responses. As a result, the pathological state becomes progressively less dependent on the original triggering factors and more reliant on internally maintained regulatory circuits.

Within this framework, endometriosis can be more accurately conceptualized as a disease driven by a sustained molecular program rather than solely by the presence of ectopic tissue. Lesions therefore represent the visible manifestation of a broader and persistent biological process, maintained through the continuous interaction of multiple regulatory systems.

This integrated model provides a mechanistic basis for disease recurrence and helps explain the limited long-term efficacy of approaches focused exclusively on anatomical removal. It also supports the need for therapeutic strategies that target the underlying biological processes, including molecular signaling, epigenetic regulation, and immune dysfunction, in order to achieve more durable disease control.

## 4. The Microenvironment: A Permissive Niche for Disease Persistence

### 4.1. The Peritoneal Microenvironment as an Active Driver of Disease

Endometriosis develops and persists within a highly dynamic and biologically active microenvironment. The peritoneal cavity represents a complex niche in which immune cells, inflammatory mediators, hormonal signals, and extracellular matrix components continuously interact to regulate cellular behavior [23,24,25,26,27]. Within this context, the microenvironment does not merely host endometriotic lesions but actively contributes to their establishment, survival, and progression [29,30].

In affected patients, peritoneal fluid is enriched with pro-inflammatory cytokines, chemokines, growth factors, and angiogenic mediators, including IL-6, TNF-α, and VEGF, which collectively promote lesion implantation, vascularization, and persistence [26,27]. These factors enhance cellular adhesion, increase matrix metalloproteinase activity, and facilitate tissue invasion. Concurrently, alterations in extracellular matrix composition and remodeling processes contribute to lesion stabilization and integration.

Importantly, this microenvironmental dysregulation is not transient. Even after surgical removal of visible lesions, the inflammatory and immunological milieu may persist, maintaining conditions that favor disease re-establishment [82,83]. This indicates that the peritoneal cavity functions as an active driver of recurrence rather than a passive background.

Within this framework, the microenvironment represents a permissive niche that integrates local and systemic signals, linking cellular reprogramming to clinical persistence and recurrence [84,85].

### 4.2. Chronic Inflammation and Self-Sustaining Feedback Loops

A hallmark of endometriosis is a persistent and dysregulated inflammatory state characterized by elevated levels of cytokines such as interleukin-6, tumor necrosis factor-α, and interleukin-1β [23,24,25,26,27]. These mediators promote proliferation, angiogenesis, and nociceptive signaling, directly contributing to both lesion maintenance and symptom generation [29,30].

This inflammatory state is sustained through self-reinforcing feedback loops. Endometriotic cells produce cytokines and chemokines that recruit immune cells, particularly macrophages and neutrophils, which in turn release additional inflammatory mediators. This bidirectional interaction establishes a chronic inflammatory circuit that supports lesion survival and progression [86].

At the molecular level, persistent activation of NF-κB signaling plays a central role, driving transcription of pro-inflammatory and anti-apoptotic genes. This pathway also interacts with estrogen signaling, amplifying estrogen-dependent proliferation and reinforcing the inflammatory phenotype [87].

In parallel, increased oxidative stress contributes to both cellular damage and adaptive responses. Reactive oxygen species (ROS) promote DNA damage, mitochondrial dysfunction, and further activation of inflammatory pathways, while also enhancing cellular resilience. Extracellular matrix remodeling and fibrosis further stabilize lesions within the peritoneal environment.

These processes generate a stable yet dynamic inflammatory ecosystem that supports persistence and recurrence, representing a key therapeutic target.

### 4.3. Immune Dysregulation and Tolerance

Immune dysfunction represents a central determinant of the permissive microenvironment in endometriosis. Under physiological conditions, ectopic endometrial cells are expected to be eliminated through effective immune surveillance. However, in affected patients, this process appears to be impaired.

Reduced natural killer (NK) cell cytotoxicity within the peritoneal environment may impair the ability of the immune system to effectively recognize and clear ectopic endometrial cells. In parallel, altered T-cell responses, including an expansion of regulatory T-cell (Treg) populations, contribute to the establishment of an immunotolerant environment [88].

Macrophages, which are highly represented in the peritoneal cavity, frequently display an M2-like phenotype characterized by pro-angiogenic, pro-fibrotic, and immunosuppressive activity. These cells produce factors such as VEGF, TGF-β, and various cytokines that support lesion growth and vascularization rather than effective clearance [65,66]. This functional shift in macrophage activity contributes to the development of a microenvironment that favors cellular survival and tissue integration [33,34].

A distinctive feature of this immune profile is its paradoxical nature. Chronic inflammation coexists with impaired cytotoxic function, resulting in a state in which immune activation does not translate into efficient elimination of ectopic tissue. Elevated levels of pro-inflammatory cytokines are maintained alongside mechanisms that actively suppress immune clearance, allowing endometriotic cells to persist and adapt over time [89].

Importantly, these immune alterations are not fully reversed by surgical intervention and may reflect broader systemic immune dysregulation. In this context, immune tolerance emerges as a key mechanism linking microenvironmental dysfunction to disease persistence and recurrence, further supporting the concept of endometriosis as a condition sustained by long-lasting biological imbalance [33,34,35,36].

### 4.4. Angiogenesis and Neurogenesis

The endometriotic microenvironment is characterized by enhanced angiogenesis and neurogenesis, both of which are essential for lesion establishment and long-term maintenance.

Increased expression of vascular endothelial growth factor (VEGF), angiopoietins, and hypoxia-inducible factors promotes the formation of new blood vessels, ensuring an adequate supply of oxygen and nutrients to ectopic lesions. Hypoxic conditions within the peritoneal environment further amplify angiogenic signaling, creating a self-reinforcing process that supports vascular network expansion and lesion survival [51,52].

At the same time, elevated levels of neurotrophic factors, including nerve growth factor (NGF), drive the development of a dense and disorganized nerve fiber network within and around endometriotic lesions. This neurogenic component is closely linked to inflammatory signaling, as pro-inflammatory cytokines contribute not only to nerve growth but also to peripheral sensitization, thereby playing a central role in the pathogenesis of chronic pelvic pain [53,54].

Angiogenesis and neurogenesis are not independent phenomena but represent closely interconnected processes that share common regulatory pathways and mediators. Their coordinated activation contributes to the formation of a neurovascular niche that enhances lesion stability, facilitates tissue integration, and promotes resistance to both physiological control mechanisms and therapeutic interventions [90].

Within this context, the development of vascular and neural networks can be viewed as a key adaptive strategy that allows endometriotic lesions to persist and function as biologically active structures within ectopic environments.

### 4.5. Endocrine-Immune Crosstalk

Endometriosis is fundamentally an estrogen-dependent condition, and the interaction between hormonal and immune pathways represents a critical component of its microenvironment. Estrogen not only promotes cellular proliferation and survival but also exerts profound immunomodulatory effects, enhancing inflammatory signaling and altering the function of multiple immune cell populations. In particular, estrogen has been shown to stimulate the production of pro-inflammatory cytokines, modulate macrophage polarization, and influence T-cell responses, thereby contributing to a persistent inflammatory milieu [22,23,24,25].

Conversely, inflammatory mediators play an active role in regulating local estrogen production. Cytokines such as interleukin-6 and tumor necrosis factor-α can upregulate aromatase expression within endometriotic tissue, leading to increased local estrogen synthesis. This local estrogen production is further amplified by the altered metabolic profile of endometriotic cells, which favors estrogen accumulation and activity within the lesion microenvironment [86,87].

The result is the establishment of a self-reinforcing feedback loop in which estrogen promotes inflammation, and inflammation in turn enhances estrogen biosynthesis and signaling. This bidirectional interaction sustains cellular proliferation, inhibits apoptosis, and reinforces immune tolerance, thereby contributing to lesion persistence and progression [88,89,90].

Importantly, this endocrine-immune crosstalk operates across both local and systemic levels, linking tissue-specific alterations to broader biological processes. It represents a key mechanism through which molecular and microenvironmental factors converge, further supporting the concept that endometriosis cannot be fully understood or effectively managed as a purely structural disease. Instead, it underscores the need for integrated approaches that address both hormonal and immune dysregulation as central drivers of disease activity.

### 4.6. The Emerging Role of the Microbiome

Recent evidence suggests that the microbiome, particularly the gut microbiota, may play a significant role in modulating both the systemic and local environment in endometriosis [40,41,42]. Alterations in microbial composition, commonly referred to as dysbiosis, have been associated with changes in immune regulation, inflammatory signaling, and estrogen metabolism, all of which are central to disease pathophysiology [91].

One of the key mechanisms linking the microbiome to endometriosis is the activity of the estrobolome, the collection of microbial genes involved in estrogen metabolism. Through the production of enzymes such as β-glucuronidase, gut bacteria can modulate the enterohepatic circulation of estrogens, increasing their deconjugation and reabsorption [40]. This process may lead to elevated systemic and local estrogen levels, thereby amplifying estrogen-driven proliferation, inflammation, and lesion persistence within endometriotic tissue [92].

Beyond hormonal regulation, the microbiome also exerts a direct influence on immune function. Dysbiosis has been associated with altered immune cell profiles, including changes in T-cell differentiation, macrophage activity, and inflammatory cytokine production. These immune alterations may contribute to the impaired clearance of ectopic endometrial cells and reinforce the state of immune tolerance characteristic of endometriosis. At the same time, microbial metabolites, such as short-chain fatty acids, may further modulate inflammatory and metabolic pathways, adding an additional layer of complexity to host–microbe interactions [93].

Importantly, the effects of the microbiome are not confined to the gut but extend systemically, influencing distant tissues through endocrine, immune, and metabolic axes. This supports the concept that microbiome-related dysregulation may contribute to both the initiation and persistence of endometriosis, interacting with genetically and epigenetically primed cells and with the permissive microenvironment described above [94].

Although this field is still evolving and causality remains to be fully established, the microbiome represents a promising area of investigation. Its integration into the pathophysiological model of endometriosis further reinforces the systemic nature of the disease and opens new perspectives for therapeutic intervention, including microbiome-targeted strategies aimed at restoring immune and hormonal homeostasis.

### 4.7. Integration: The Microenvironment as a Sustaining System

The components of the microenvironment, including chronic inflammation, immune dysregulation, angiogenesis, hormonal signaling, and microbial influences, are deeply interconnected and operate as part of an integrated system that sustains endometriosis. Rather than acting as independent contributors, these elements interact continuously through bidirectional signaling pathways, creating a dynamic network that supports lesion survival, growth, and adaptation [23,24,25,26,27,28,29,30,33,34,35,36].

This system is not necessarily disrupted by surgical intervention. While surgery effectively removes visible lesions and restores anatomical relationships, it does not directly address the underlying inflammatory, immune, and endocrine networks that drive disease persistence. As a result, the biological context in which endometriosis develops may remain largely unchanged, preserving a permissive environment that allows residual or newly implanted cells to survive, proliferate, and ultimately re-establish lesions [95,96,97].

In this setting, the persistence of chronic inflammatory signaling, together with impaired immune surveillance and sustained hormonal stimulation, contributes to the maintenance of a pro-survival niche. These factors not only facilitate cellular implantation and growth but also reinforce the altered phenotype of endometriotic cells, further stabilizing the disease process over time [98].

Within this framework, the microenvironment can be more accurately conceptualized as a biological ecosystem that actively maintains and reinforces the reprogrammed state of endometriotic cells. By linking molecular alterations to tissue-level and systemic dynamics, this ecosystem bridges the gap between cellular pathology and clinical recurrence, providing a coherent explanation for the persistence and resilience of the disease (Table 2) [99,100].

## 5. A Systemic Disease Model of Endometriosis

### 5.1. From a Localized Disorder to a Systemic Condition

The traditional view of endometriosis as a localized pelvic disease is increasingly insufficient to explain its clinical behavior, including recurrence, multifocality, and heterogeneity in symptom presentation [101,102]. Although the identification and surgical removal of ectopic lesions have long represented the cornerstone of management, this lesion-centered perspective does not fully account for the persistence of symptoms and the frequent relapse observed even after apparently complete excision [31,32]. The evidence discussed in previous sections suggests that endometriosis extends beyond the simple presence of ectopic tissue and instead reflects a complex interplay of molecular, cellular, and environmental processes that operate across multiple biological levels [40,41].

Within this broader framework, endometriosis can be more accurately interpreted as a systemic condition, in which altered cellular programming, immune dysfunction, and endocrine imbalance interact dynamically over time. Endometriotic cells exhibit stable molecular alterations that enable them to survive, proliferate, and evade physiological control mechanisms, while the surrounding microenvironment provides a permissive context that sustains these pathological features. At the same time, systemic factors such as hormonal regulation, immune system dynamics, and metabolic influences contribute to shaping disease behavior both locally and at distant sites [103,104].

This integrated perspective helps explain several key features of endometriosis that remain poorly understood within a purely anatomical model. These include the variability in disease onset, the wide spectrum of clinical presentations, the occurrence of lesions in multiple anatomical compartments, and the unpredictable response to both surgical and medical treatments. It also provides a coherent framework to understand why symptoms may persist even in the absence of detectable lesions, suggesting that the underlying biological alterations extend beyond what can be visualized or removed surgically [105].

Within this context, pelvic lesions should be regarded not as the disease itself, but as the visible manifestation of a more widespread and persistent pathological process. This distinction is critical, as it shifts the focus from a static, lesion-based interpretation toward a dynamic, system-level understanding of endometriosis, with important implications for both research and clinical management.

### 5.2. Integration of Cellular, Microenvironmental, and Systemic Factors

A systemic disease model of endometriosis emerges from the integration of three interrelated and mutually reinforcing components. Intrinsic cellular reprogramming, characterized by stable alterations in gene expression, epigenetic regulation, and intracellular signaling pathways, confers a survival advantage and resistance to apoptosis in endometriotic cells [105]. At the same time, a permissive microenvironment shaped by chronic inflammation, immune tolerance, angiogenesis, and endocrine-immune crosstalk supports lesion implantation, maintenance, and progression [14,15,16]. In parallel, systemic modulators, including hormonal regulation, immune system dynamics, metabolic factors, and potentially the microbiome, exert both local and distant effects that further influence disease behavior [20,21,22,40].

Importantly, these components do not operate in isolation, but interact dynamically, forming a self-reinforcing network that sustains disease activity over time. Continuous bidirectional communication between reprogrammed cells and their surrounding environment amplifies pathological signaling and stabilizes the disease state [35,36,37].

Within this framework, surgical removal of lesions addresses only the anatomical manifestation of the disease, while leaving the underlying molecular, microenvironmental, and systemic drivers largely intact. The persistence of this integrated network provides a mechanistic explanation for recurrence and highlights the limitations of therapeutic strategies that fail to target the broader biological context of endometriosis.

### 5.3. Endometriosis as a Self-Sustaining Biological Network

Within this paradigm, endometriosis can be conceptualized as a self-sustaining biological network rather than a static anatomical condition. Once reprogrammed, endometriotic cells continuously interact with their surrounding environment, generating dynamic feedback loops that reinforce inflammation, hormonal dysregulation, and immune evasion [23,24,25,26,27,28,29,30].

These interconnected mechanisms establish a stable pathological state that is inherently resilient to interventions targeting a single component of the system. For example, surgical excision may effectively remove the anatomical manifestation of the disease but does not address the underlying molecular and systemic drivers that sustain its activity. Likewise, hormonal therapies can modulate endocrine signaling but may fail to fully reverse epigenetic alterations or restore immune competence [106].

This network-based perspective is consistent with emerging models of other chronic inflammatory and immune-mediated disorders, in which disease persistence is driven by coordinated dysregulation across multiple biological domains. In this context, endometriosis should be viewed as a dynamic and adaptive system, capable of maintaining its pathological state through continuous interactions between cellular, microenvironmental, and systemic factors [107].

Within this framework, disease activity should be conceptualized along a spectrum ranging from overt lesion recurrence to subclinical biological persistence. This includes patients with persistent symptoms in the absence of visible lesions, in whom molecular, inflammatory, and neuroimmune mechanisms may remain active despite surgical clearance.

### 5.4. Temporal Dynamics and Disease Evolution

A systemic model also enables a more nuanced understanding of disease evolution over time. Endometriosis may originate from early-life or developmental factors, including genetic predisposition and epigenetic priming, and subsequently evolve under the influence of cyclical hormonal activity, immune system maturation, and environmental exposures [108].

Within this dynamic framework, the interplay between intrinsic cellular programming and external modulators shapes not only disease initiation but also its progression and response to treatment [14,15,16,17,18]. The timing and intensity of these interactions may critically influence the trajectory of the disease, particularly during key biological windows such as puberty and early reproductive life [16,31,32].

This perspective provides a coherent explanation for the marked heterogeneity observed among patients, including differences in age at onset, anatomical distribution of lesions, symptom severity, and risk of recurrence. It also supports the concept that endometriosis is not a static condition but a continuously evolving disorder, in which biological, environmental, and temporal factors converge to define individual disease phenotypes [109,110,111,112].

### 5.5. Clinical Implications of a Systemic Model

Reframing endometriosis as a biologically sustained and potentially systemic condition has important implications for clinical management. While surgery remains an essential therapeutic option in selected patients, particularly in cases of severe anatomical distortion, infertility, or organ involvement, the present framework suggests that therapeutic strategies should extend beyond the removal or suppression of visible lesions and increasingly address the underlying biological mechanisms sustaining disease activity. In this context, endometriosis should not be viewed exclusively as a surgically manageable condition, but rather as a heterogeneous disorder that, in some patients, may require long-term and mechanism-based management strategies [45,46,47].

Importantly, disease persistence may not always manifest as overt anatomical recurrence. A proportion of patients continue to experience pain and functional impairment despite apparently complete surgical excision and in the absence of detectable lesions at imaging or repeat surgery. This clinical scenario suggests that persistent inflammatory signaling, neuroimmune activation, microscopic disease foci, or sustained molecular alterations may remain biologically active even after macroscopic clearance of lesions.

Within this perspective, greater emphasis may need to be placed on early phenotype-adapted medical strategies, particularly in patients considered at higher risk of recurrence or persistent disease activity. Chronic inflammatory pathway modulation may help disrupt the self-sustaining inflammatory loops that support lesion survival and symptom generation [105]. At the same time, restoring immune competence and reducing the permissive inflammatory microenvironment may contribute to limiting the persistence of ectopic cellular activity. Addressing hormonal dysregulation, especially the imbalance between estrogen-driven signaling and progesterone resistance, remains a cornerstone of treatment, although ideally integrated within a broader and more individualized therapeutic framework [106,107,108,109].

This evolving model also has implications for patients experiencing recurrence or persistent symptoms despite apparently adequate surgical treatment. In these cases, repeated surgery alone may not fully address the underlying biological drivers of disease activity. Instead, long-term suppression strategies and integrated management approaches targeting hormonal, inflammatory, immune, and potentially molecular pathways may become increasingly relevant. In this regard, endometriosis may share important conceptual similarities with other chronic inflammatory disorders characterized by fluctuating disease activity and long-term biological persistence.

Importantly, emerging evidence highlights the potential future role of therapies targeting epigenetic and molecular alterations underlying the persistent reprogramming of endometriotic cells. Interventions aimed at modulating gene expression patterns, inflammatory signaling, immune dysfunction, or intracellular pathways may offer novel opportunities to interfere with the “molecular memory” potentially contributing to persistence and recurrence [110,111].

Taken together, these considerations support the need for a multidimensional and individualized therapeutic strategy combining surgical, medical, and potentially future targeted approaches. Such an integrated framework may help achieve more durable disease control, reduce recurrence risk, improve symptom management, and better address the heterogeneous clinical behavior of endometriosis, particularly in patients with aggressive, recurrent, or treatment-resistant phenotypes.

### 5.6. Toward a Paradigm Shift

**Figure 1:**
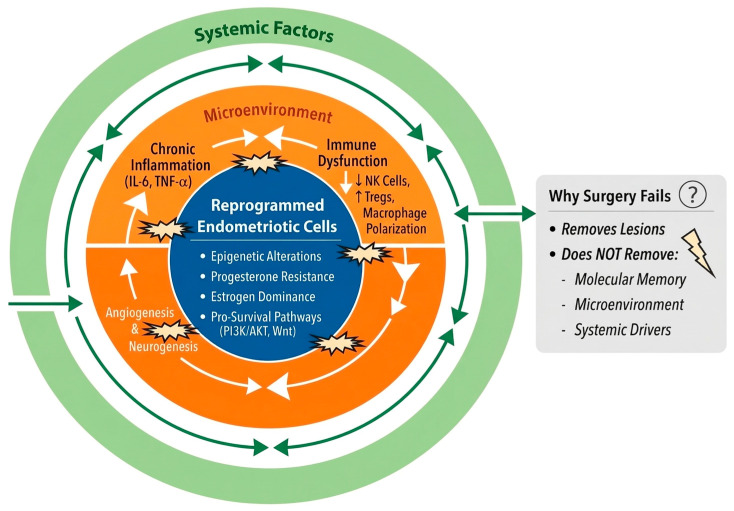
Endometriosis as a Reprogrammed Systemic Disease: Integration of Cellular, Microenvironmental, and Systemic Drivers. Schematic representation of endometriosis as a reprogrammed systemic disease. Reprogrammed endometriotic cells, characterized by epigenetic alterations, progesterone resistance, estrogen dominance, and activation of pro-survival signaling pathways, interact dynamically with the surrounding microenvironment, including chronic inflammation, immune dysfunction, and angiogenesis/neurogenesis. These interactions are further modulated by systemic factors, creating a self-sustaining network that supports disease persistence and recurrence. Surgical excision removes visible lesions but does not eliminate the underlying molecular, microenvironmental, and systemic drivers of disease. (ER, estrogen receptor; PR, progesterone receptor; IL, interleukin; TNF, tumor necrosis factor; NK, natural killer; Treg, regulatory T cell; PI3K, phosphoinositide 3-kinase; AKT, protein kinase B; Wnt, wingless-related integration site; VEGF, vascular endothelial growth factor).

The integration of molecular reprogramming, microenvironmental dynamics, and systemic regulation supports a paradigm shift in the understanding of endometriosis. Rather than a condition defined solely by the presence of ectopic tissue, endometriosis should be viewed as a persistent, system-level disorder driven by coordinated biological dysregulation (Figure 1 and Table 3) [112]. 

This evolving framework challenges the traditional reductionist model that has long guided both diagnosis and treatment. While the lesion-based approach has provided important clinical and surgical insights, it fails to fully capture the complexity of the disease, particularly in explaining recurrence, heterogeneity in clinical presentation, and variable responses to therapy. In contrast, a systems-level perspective recognizes that endometriosis arises from the interaction of multiple biological layers, including genetically and epigenetically altered cells, a permissive inflammatory and immune microenvironment, and broader systemic influences [113].

Within this paradigm, endometriotic lesions are no longer interpreted as isolated pathological entities, but rather as dynamic manifestations of an underlying and self-sustaining biological network. The concept of persistent “molecular memory,” maintained through epigenetic mechanisms, provides a mechanistic explanation for the long-term stability of the disease phenotype and its ability to recur even after apparently complete surgical excision. At the same time, the contribution of immune tolerance, chronic inflammation, and endocrine-immune crosstalk reinforces the idea that disease persistence is not confined to local tissue but is supported by a system-wide dysregulation [112,113,114].

Importantly, this conceptual shift has direct implications for clinical management. It suggests that therapeutic strategies should move beyond targeting visible lesions and instead aim to modulate the underlying biological processes that sustain the disease. This includes the development of interventions directed at inflammatory pathways, immune function, hormonal regulation, and epigenetic mechanisms. Such an approach aligns with the broader movement toward precision medicine, in which treatment is tailored to the individual biological profile of the patient rather than solely to anatomical findings [115].

Moreover, adopting a systemic framework opens new avenues for research, including the identification of circulating biomarkers, the exploration of microbiome-host interactions, and the integration of multi-omics data to better characterize disease subtypes. It also encourages the development of longitudinal models that account for disease evolution over time, rather than static snapshots based on surgical findings [116].

Ultimately, redefining endometriosis through this integrated lens not only enhances our understanding of its pathophysiology but also provides a more coherent foundation for the development of innovative and durable therapeutic strategies. This paradigm shift represents a necessary step toward overcoming the limitations of current approaches and improving long-term outcomes for affected patients.

### 5.7. Limitations of Current Evidence

Despite the growing body of evidence supporting the potential role of molecular reprogramming in endometriosis, several important limitations must be acknowledged. First, a substantial proportion of the currently available molecular, epigenetic, transcriptomic, and immunological data derives from in vitro studies, experimental models, or relatively small and heterogeneous patient cohorts. Differences in study design, tissue sampling, disease phenotype classification, hormonal exposure, and analytical methodologies may significantly influence reported findings and limit direct comparability across studies. Consequently, the biological relevance and reproducibility of some molecular alterations remain incompletely defined.

In addition, although numerous studies have identified associations between epigenetic dysregulation, inflammatory signaling, hormonal imbalance, and disease persistence, definitive causal relationships have not yet been fully established [112]. Most available evidence remains observational or cross-sectional in nature, while prospective longitudinal studies specifically evaluating the temporal relationship between molecular alterations, symptom persistence, and recurrence after treatment are still limited. As a result, it remains difficult to determine whether some of the observed biological abnormalities represent primary drivers of disease development, adaptive responses to the ectopic microenvironment, or secondary consequences of chronic inflammation and tissue injury.

Similarly, the interpretation of immune and inflammatory alterations requires caution. While chronic inflammation and immune dysregulation are consistently associated with endometriosis, the directionality of these interactions remains incompletely understood. Immune dysfunction may contribute to lesion establishment and persistence, but may also arise as a consequence of ongoing disease activity, creating a complex bidirectional relationship that is not yet fully clarified.

The role of the microbiome also remains largely exploratory [42]. Although increasing evidence supports a potential interaction between gut microbial composition, estrogen metabolism, immune regulation, and inflammatory signaling, current data are predominantly associative and characterized by methodological heterogeneity. Interventional studies demonstrating direct causality or clinically meaningful therapeutic benefit remain limited, and the exact contribution of host–microbiome interactions to disease persistence has yet to be clearly established.

Importantly, while the present review emphasizes the concept of biological persistence, this should not minimize the established importance of surgical quality and completeness of excision. International guidelines consistently demonstrate that high-quality surgery performed by experienced surgeons significantly improves symptom control and reduces recurrence risk. Incomplete excision, residual disease, and disease severity undoubtedly remain important contributors to recurrence in a substantial proportion of patients. Accordingly, the present framework should not be interpreted as opposing surgical management, but rather as an attempt to integrate surgical and biological perspectives into a broader understanding of disease behavior [32].

Another important limitation relates to the heterogeneity of endometriosis itself. Endometriosis likely represents a spectrum of biologically distinct phenotypes rather than a single uniform disorder. Differences in lesion type, anatomical distribution, symptom profile, hormonal responsiveness, genetic background, immune regulation, reproductive impact, and environmental exposures may all influence disease behavior and treatment response. Consequently, the concept of molecular reprogramming proposed in this review should not be interpreted as universally applicable to all patients with endometriosis.

Instead, it is possible that persistent molecular and systemic alterations characterize only a subset of individuals, particularly those with recurrent disease, persistent pain despite apparently adequate treatment, treatment-resistant phenotypes, or discordance between lesion burden and symptom severity. Moreover, disease persistence may not necessarily manifest exclusively as overt anatomical recurrence. In some patients, persistent inflammatory, neuroimmune, hormonal, or epigenetic activity may continue even in the absence of detectable lesions at imaging or repeat surgery, potentially contributing to chronic symptoms and impaired quality of life.

Finally, many of the concepts discussed in this review, including “molecular memory,” systemic dysregulation, and persistent biological activity beyond visible disease, remain evolving theoretical frameworks rather than definitively established pathogenetic models. Further large-scale translational studies integrating molecular profiling, longitudinal clinical follow-up, imaging, and therapeutic response data will be essential to clarify the extent to which these mechanisms contribute to disease initiation, persistence, recurrence, and clinical heterogeneity.

## 6. Implications of Molecular Reprogramming on Fertility

### 6.1. Endometrial Receptivity and Implantation

Endometrial receptivity represents a finely regulated and temporally restricted process that is critically dependent on coordinated hormonal, molecular, and immune signaling. In endometriosis, molecular reprogramming profoundly disrupts this process, contributing to implantation failure and infertility [117].

One of the most consistently reported alterations involves aberrant epigenetic regulation of genes essential for endometrial differentiation and receptivity. In particular, hypermethylation of the HOXA10 promoter has been associated with reduced gene expression, impairing uterine receptivity and decidualization capacity. This alteration is closely linked to progesterone resistance, a hallmark of endometriosis, which disrupts the transcriptional response required for the establishment of the implantation window [118,119].

At the cellular level, impaired progesterone signaling leads to defective expression of adhesion molecules, including integrins (αvβ3), leukemia inhibitory factor (LIF), and other implantation-related mediators. These molecules are essential for embryo attachment and invasion, and their dysregulation results in a suboptimal uterine environment [119,120].

Furthermore, the dialog between the embryo and maternal endometrium is altered. The endometrial immune environment, characterized by increased inflammatory cytokines (e.g., IL-6, TNF-α) and altered immune cell populations, interferes with the delicate balance required for successful implantation [119,120,121]. Dysregulated interactions between trophoblast cells and uterine immune cells further compromise embryo acceptance.

These findings support the concept that molecular reprogramming not only sustains ectopic lesion persistence but also directly impairs reproductive competence at the level of the eutopic endometrium.

### 6.2. Peritoneal Environment and Gamete Quality

The peritoneal environment in endometriosis is characterized by chronic inflammation and oxidative stress, which exert detrimental effects on both oocytes and spermatozoa [122].

Elevated levels of pro-inflammatory cytokines and reactive oxygen species (ROS) in peritoneal fluid have been shown to impair oocyte quality by disrupting meiotic spindle formation, mitochondrial function, and cytoplasmic maturation. Oxidative stress can induce DNA damage and alter intracellular signaling pathways critical for oocyte competence [123].

Similarly, sperm function is negatively affected by exposure to inflammatory mediators and oxidative stress. Reduced sperm motility, increased DNA fragmentation, and impaired fertilization capacity have been reported in the context of endometriosis-associated peritoneal fluid [124].

In addition, chronic inflammation influences folliculogenesis. Altered cytokine profiles within the ovarian microenvironment may impair granulosa cell function and disrupt follicular development, ultimately compromising oocyte competence [125].

These observations highlight that the effects of molecular reprogramming extend beyond ectopic lesions, influencing the broader reproductive environment and reducing the likelihood of successful fertilization.

### 6.3. Impact on Ovarian Reserve

Endometriosis, particularly in the form of ovarian endometriomas, is associated with a progressive decline in ovarian reserve. This phenomenon is mediated by a combination of molecular, inflammatory, and mechanical mechanisms [126].

Endometriomas are characterized by a microenvironment rich in oxidative stress and inflammatory mediators, which can induce follicular atresia and damage the surrounding ovarian cortex. Increased levels of ROS contribute to DNA damage, mitochondrial dysfunction, and apoptosis within follicular cells [127].

Fibrotic changes and chronic inflammation further compromise ovarian tissue integrity, while mechanical compression exerted by cystic lesions reduces vascularization and disrupts normal follicular architecture [128,129,130].

Importantly, surgical excision of endometriomas, although effective for symptom relief, may exacerbate the decline in ovarian reserve. The removal of cyst walls can inadvertently lead to the loss of healthy ovarian tissue, highlighting a well-recognized clinical paradox.

Within the framework of molecular reprogramming, these findings suggest that ovarian damage is not solely structural but also driven by persistent biological alterations that continue to affect ovarian function over time [131].

### 6.4. Implications for Assisted Reproductive Technologies (ART)

Endometriosis has been associated with variable outcomes in assisted reproductive technologies (ART), including IVF and ICSI [132].

While fertilization rates may be relatively preserved, several studies have reported reduced implantation and pregnancy rates, particularly in moderate-to-severe disease. These outcomes are likely influenced by both impaired endometrial receptivity and altered oocyte quality [133].

The concept of molecular reprogramming provides a unifying framework to interpret these findings. Persistent alterations in hormonal signaling, immune function, and epigenetic regulation may compromise both embryo quality and uterine receptivity, even in controlled ART settings [134].

This perspective has important clinical implications. It supports the need for individualized treatment strategies, including careful timing of surgery, optimization of ovarian stimulation protocols, and potential integration of adjunctive therapies targeting inflammation or immune dysfunction.

Moreover, understanding the molecular basis of endometriosis-related infertility may facilitate the development of novel biomarkers to predict ART outcomes and guide personalized therapeutic approaches [135].

## 7. Conclusions

Endometriosis has long been approached as a localized gynecological disorder amenable to the surgical removal of ectopic lesions. However, the persistence of symptoms and the recurrence of disease following apparently complete excision challenge this reductionist view and point toward a more complex underlying biological framework [31,32,53,54,55]. Importantly, clinical persistence does not always manifest as overt lesion recurrence, as a proportion of patients continue to experience pain and functional impairment even in the absence of detectable disease.

The evidence reviewed in this work suggests that endometriosis may be sustained by an integrated network of alterations, including intrinsic cellular reprogramming, epigenetic regulation, chronic inflammation, immune dysfunction, and endocrine imbalance [14,15,16,17,18]. These mechanisms interact dynamically, creating a biological context capable of maintaining disease activity beyond the mere anatomical presence of lesions and not fully reversed by surgical intervention [19,20,21,22].

Within this perspective, endometriosis can be more appropriately interpreted, at least in a subset of patients, as a persistent and biologically sustained condition in which lesions represent the visible manifestation of a broader and self-maintaining process [24,25,26]. In this context, disease persistence may encompass a spectrum ranging from macroscopic recurrence to ongoing subclinical or microscopic activity, potentially contributing to symptom generation even in the absence of identifiable lesions. This framework offers a plausible explanation for the heterogeneity of clinical outcomes and highlights the limitations of therapeutic strategies focused exclusively on lesion removal [28,29,30].

Recognizing that these mechanisms are unlikely to be uniformly present across all patients, but may instead characterize specific phenotypes, has important implications for both research and clinical practice. It underscores the need to move beyond a purely anatomical approach and to adopt integrated, mechanism-based strategies targeting the molecular, inflammatory, and immunological drivers of disease persistence.

Ultimately, viewing endometriosis through this more nuanced and integrative lens may contribute to a better understanding of its heterogeneous nature and support the development of more effective, personalized, and durable therapeutic approaches [45,46,47,136,137].

## Figures and Tables

**Table 1:** Molecular Reprogramming Landscape of Endometriotic Cells: Key Mechanisms and Functional Implications.

Domain	Specific Alterations	Underlying Mechanisms	Functional Consequences	Supporting Evidence (Type)	Therapeutic Implications	References
Hormonal signaling	(↑) ERβ/ERα imbalance; (↓) PR expression	Aberrant receptor expression; altered steroid metabolism ((↑) aromatase)	Estrogen dominance, progesterone resistance, sustained proliferation	Transcriptomic, IHC, functional studies	Progestins resistance; rationale for GnRH antagonists	[24,78,79,80]
Gene expression	Dysregulation of ESR1, WNT4, inflammatory genes	Transcriptional reprogramming	Enhanced adhesion, invasion, implantation	RNA-seq, GWAS, comparative endometrium studies	Targeting signaling pathways	[14,15,16,17,18,19]
Epigenetic regulation	DNA hyper/hypomethylation; histone modifications; ncRNAs	Epigenetic silencing/activation of key genes (e.g., HOXA10, PGR)	Stable “molecular memory” phenotype	Epigenome-wide association studies	Epigenetic modulators (future therapies)	[20,21,22,81]
Cell survival	Activation of PI3K/AKT/mTOR; Wnt/β-catenin	Pro-survival signaling, anti-apoptotic pathways	Resistance to apoptosis, enhanced growth	In vitro/in vivo models	Targeted inhibitors (PI3K, mTOR)	[17,18,19,82]
Inflammatory signaling	(↑) IL-6, TNF-α, NF-κB activation	Cytokine-driven transcriptional activation	Chronic inflammation, angiogenesis, pain	Peritoneal fluid studies, molecular assays	Anti-inflammatory therapies	[27,28,29,30]
Immune evasion	(↓) NK cytotoxicity; macrophage M2 polarization	Immune tolerance mechanisms	Persistence of ectopic cells	Immunological profiling studies	Immune-targeted strategies	[31,32,33,34,69]
Angiogenesis	(↑) VEGF, HIF-1α	Hypoxia-driven signaling	Neovascularization of lesions	Histological and molecular data	Anti-angiogenic approaches	[27,50,51,52]

ER, estrogen receptor; PR, progesterone receptor; IL, interleukin; TNF, tumor necrosis factor; NF-κB, nuclear factor kappa B; PI3K, phosphoinositide 3-kinase; AKT, protein kinase B; mTOR, mechanistic target of rapamycin; Wnt, wingless-related integration site; ncRNA, non-coding RNA; HIF, hypoxia-inducible factor; VEGF, vascular endothelial growth factor. (↑) indicates upregulation/increase; (↓) indicates downregulation/decrease.

**Table 2:** Microenvironmental and Systemic Drivers of Endometriosis: Mechanisms Underlying Disease Persistence.

Component	Key Mediators/ Pathways	Mechanistic Role	Interaction with Cellular Reprogramming	Persistence After Surgery	Clinical Relevance	References
Peritoneal inflammation	IL-6, TNF-α, IL-1β, ROS	Sustains inflammatory loop and lesion growth	Reinforces NF-κB signaling and gene expression changes	Yes	Pain, disease progression	[27,28,29,30]
Immune dysfunction	NK cells (↓), Tregs (↑), macrophage M2 phenotype	Impaired clearance of ectopic cells	Supports immune escape of reprogrammed cells	Yes	Recurrence risk	[31,32,33,34,66,67,68,69]
Angiogenesis	VEGF, angiopoietins	Promotes vascular supply to lesions	Supports survival of invasive cells	Yes	Lesion maintenance	[50,51,52]
Neurogenesis	NGF, nerve fiber density	Pain sensitization	Linked to inflammatory signaling	Yes	Chronic pelvic pain	[53,54]
Endocrine system	Estrogen, aromatase, ERβ	Local estrogen production	Enhances proliferation and inflammation	Yes	Hormone- dependent persistence	[23,24,25,26]
Endocrine–immune crosstalk	Estrogen–cytokine feedback loops	Amplifies inflammation and immune modulation	Stabilizes reprogrammed phenotype	Yes	Therapy resistance	[23,24,25,27]
Microbiome (gut)	Estrobolome, dysbiosis	Modulates estrogen metabolism and immunity	Indirectly sustains hormonal imbalance	Emerging evidence	Systemic modulation target	[40,41,42]
Extracellular matrix	Fibrosis, TGF-β signaling	Tissue remodeling, lesion anchoring	Facilitates invasion	Yes	Deep infiltrating disease	[19,35,37]

IL, interleukin; TNF, tumor necrosis factor; ROS, reactive oxygen species; NK, natural killer; Treg, regulatory T cell; VEGF, vascular endothelial growth factor; NGF, nerve growth factor; ER, estrogen receptor. (↑) indicates upregulation/increase; (↓) indicates downregulation/decrease.

**Table 3:** Lesion-Based versus Reprogrammed Systemic Model of Endometriosis: A Conceptual Comparison.

Dimension	Lesion-Based Model	Reprogrammed Systemic Model	Implications for Practice	References
Disease definition	Anatomical presence of ectopic tissue	Persistent multi-level biological disorder	Shift toward systems biology	[1,2,3,4,38,39]
Pathogenesis	Retrograde menstruation, implantation	Cellular reprogramming + microenvironment + systemic factors	Multifactorial origin	[12,13,14,15,16,35,36,37]
Cellular phenotype	Normal endometrial cells	Genetically/epigenetically altered cells	Target cellular behavior	[14,15,16,20,21,22]
Role of epigenetics	Minimal or absent	Central (molecular memory)	New therapeutic targets	[20,21,22,111,112]
Inflammation	Secondary consequence	Primary driver	Anti-inflammatory strategies	[27,28,29,30]
Immune system	Limited involvement	Key regulator (tolerance, escape)	Immunomodulation	[31,32,33,34]
Hormonal role	Estrogen-dependent growth	Integrated endocrine–immune dysregulation	Personalized hormonal therapy	[23,24,25,26]
Recurrence explanation	Incomplete excision	Persistent molecular and systemic drivers	Long-term management required	[6,7,8,9,10,11,12,13]
Surgery	Potentially curative	Symptom control but not curative	Combine with systemic therapy	[6,7,8,43,44,45]
Therapeutic strategy	Surgical ± hormonal suppression	Multimodal (molecular, immune, endocrine)	Precision medicine approach	[43,44,45,72,73]
Disease nature	Local	Systemic, dynamic network	Redefinition of disease	[38,39]

The term “molecular memory” refers to relatively persistent epigenetic and transcriptional alterations potentially contributing to the maintenance of the pathological phenotype over time.

## Data Availability

The present review was based on published articles. All summary data generated during this study are included in this published article. Raw data used for the analyses are available presented in the original reviewed articles.

## Abbreviations

ER	estrogen receptor
PR	progesterone receptor
ERβ	estrogen receptor beta
ERα	estrogen receptor alpha
IL	interleukin
TNF	tumor necrosis factor
NF-κB	nuclear factor kappa B
PI3K	phosphoinositide 3-kinase
AKT	protein kinase B
mTOR	mechanistic target of rapamycin
Wnt	wingless-related integration site
TGF-β	transforming growth factor beta
VEGF	vascular endothelial growth factor
HIF-1α	hypoxia-inducible factor 1 alpha
NK	natural killer
Treg	regulatory T cell
ROS	reactive oxygen species
ECM	extracellular matrix
ncRNA	non-coding RNA
HOXA10	homeobox A10
ESR1	estrogen receptor 1
PGR	progesterone receptor gene
